# Associations of Cumulative Conicity Index and Its Trajectories with Stroke Among Chinese Older Adults: A Prospective Cohort Study

**DOI:** 10.3390/healthcare14213741

**Published:** 2026-11-03

**Authors:** Ying Li, Chuangsen Fang, Xiaohan Zhao, Xiaoshuo Li, Deping Liu

**Affiliations:** 1 Department of Cardiology, Beijing Hospital, National Center of Gerontology, Institute of Geriatric Medicine, Chinese Academy of Medical Sciences, Beijing 100730, China; 2411110751@stu.pku.edu.cn (Y.L.); b2025021013@student.pumc.edu.cn (X.Z.); 2411210662@stu.pku.edu.cn (X.L.); 2 Peking University Fifth School of Clinical Medicine, Beijing 100730, China; 3 Department of Respiratory, Beijing Hospital, National Center of Gerontology, Institute of Geriatric Medicine, Chinese Academy of Medical Sciences, Beijing 100730, China; 18811736355@163.com

**Keywords:** conicity index, stroke, obesity, risk, CHARLS

## Abstract

**Highlights:**

**What are the main findings?**

• High levels of cumulative conicity index are associated with an increased risk of stroke among Chinese middle-aged and older adults.
• Longitudinal trajectories of the conicity index reveal heterogeneity in the risk of stroke across different obesity phenotypes.

**Abstract:**

**Background:** The conicity index (C-index) is an anthropometric indicator of body fat distribution. This study aimed to explore the associations of cumulative C-index and its trajectories with self-reported stroke among Chinese adults. **Methods:** Data were obtained from the China Health and Retirement Longitudinal Study. Exposures included cumulative C-index and C-index trajectories during 2011–2015. The outcome was self-reported incident stroke. Cumulative C-index was categorized by tertiles, and group-based trajectory modeling was used to identify distinct C-index trajectories. We used Cox proportional hazards regression models to investigate the associations of cumulative C-index and C-index trajectories with stroke. **Results:** A total of 6179 participants were included in this study. During a median follow-up of 4.6 years, incident stroke occurred in 489 (7.91%) participants. After adjusting for demographic characteristics and lifestyle-related factors, participants in the highest tertile of cumulative C-index (T3) had a 72% higher risk of stroke than those in the lowest tertile (T1) (HR = 1.72, 95% CI: 1.37–2.16, *p* < 0.001). Across C-index trajectory groups, individuals with a persistently high C-index had an 80% higher risk of stroke (HR = 1.80, 95% CI: 1.36–2.39, *p* < 0.001) than those with a persistently low C-index. After further adjustment for metabolic comorbidities and laboratory markers, the associations were somewhat attenuated but remained statistically significant. **Conclusions:** A higher cumulative C-index and persistently high C-index were associated with a significantly elevated risk of self-reported incident stroke among middle-aged and older Chinese adults.

## 1. Introduction

Stroke is a leading cause of death and disability, posing a substantial threat to population health and representing a major public health problem worldwide [1,2]. Efforts should be made to identify risk factors to inform stroke prevention and management. Notably, obesity is a prominent risk factor for stroke and is associated with poor prognosis in stroke patients. Previous studies have primarily relied on body mass index (BMI) to assess obesity status [3,4]. However, BMI cannot fully capture body fat distribution and does not effectively distinguish fat mass from muscle mass. Recently, researchers have explored more accurate anthropometric indicators of obesity, including the body roundness index (BRI), a body shape index (ABSI), the conicity index (C-index), the relative fat mass (RFM), and the waist-to-height ratio (WHtR) [5,6,7,8,9,10]. The C-index, which incorporates waist circumference (WC), weight, and height, can reflect central adiposity [11,12].

Previous studies have shown that the C-index is associated with hyperuricemia, diabetes, and other metabolic diseases [9,13,14]. Zhang et al. reported an association between the C-index and risk of stroke among rural Chinese adults [15]. Nevertheless, a single C-index measurement cannot capture long-term obesity-related risk over time. Therefore, this study aimed to investigate the associations of cumulative C-index and C-index trajectories with stroke risk in middle-aged and older Chinese adults, thereby providing new insights for stroke prevention and treatment.

## 2. Materials and Methods

### 2.1. Study Participants

This prospective study recruited all participants from the China Health and Retirement Longitudinal Study (CHARLS), a nationally representative survey of Chinese adults aged 45 and above. The baseline survey was conducted in 2011–2012 (Wave 1), with follow-up interviews in 2013 (Wave 2), 2015 (Wave 3), 2018 (Wave 4), and 2020 (Wave 5) [16]. CHARLS was approved by the Institutional Review Board at Peking University (IRB00001052-11015), and all participants provided written informed consent.

**Figure 1:**
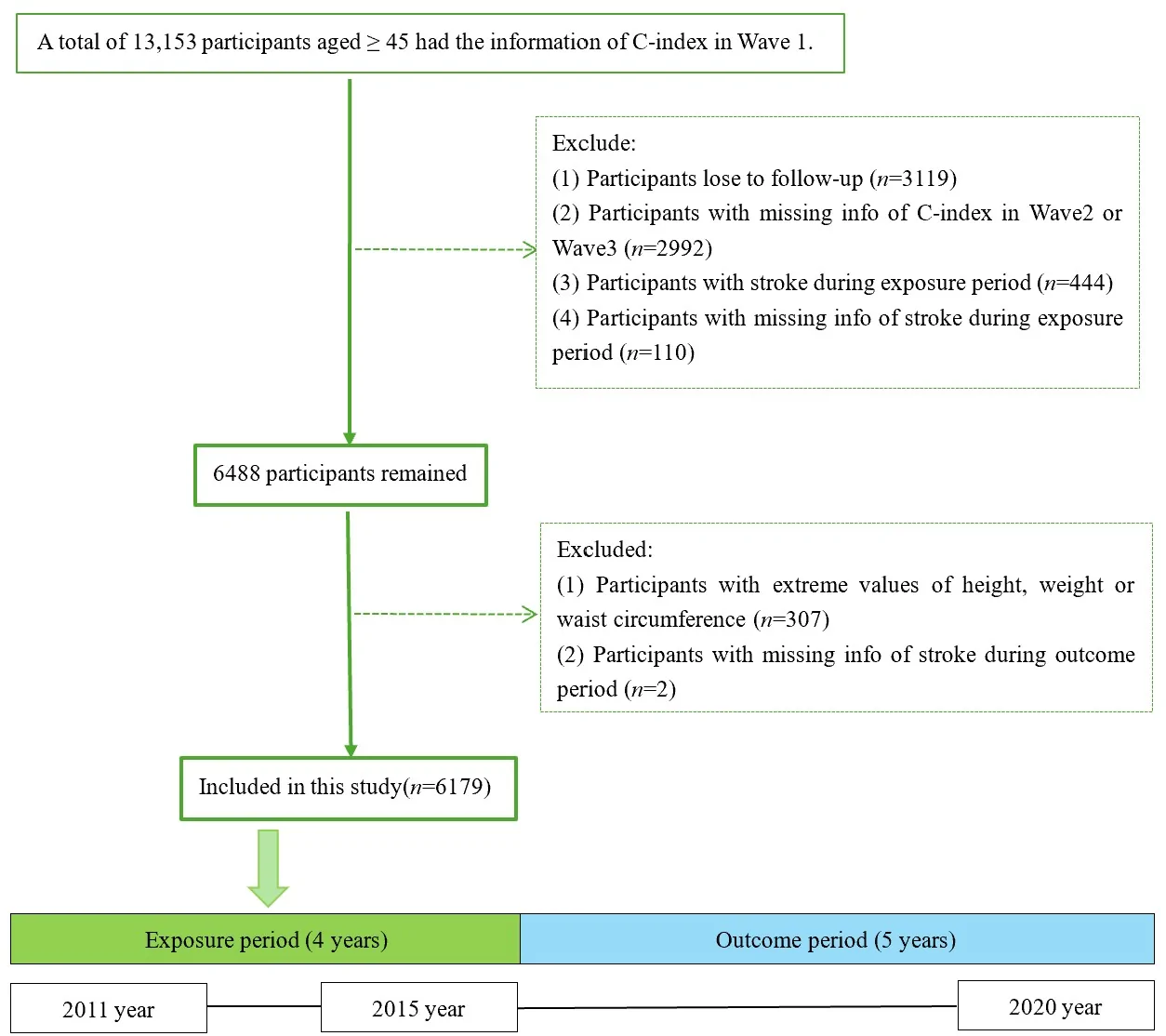
Flow chart of participant screening.

Participants were recruited beginning in 2011. The primary variable, the C-index, was calculated using data collected from 2011 to 2015 (Waves 1–3). We ascertained stroke outcomes from survey data collected in 2018 (Wave 4) and 2020 (Wave 5). Participants were eligible if they completed all three consecutive surveys across Waves 1–3. We excluded participants who experienced a stroke during the exposure period (Waves 1–3). Specifically, we initially identified 13,153 participants aged ≥45 years from the CHARLS cohort who had Wave 1 data available for C-index calculation. A total of 6974 participants were excluded for the following reasons: (1) Participants who were lost to follow-up (*n* = 3119); (2) Participants with missing C-index data in Wave 2 or Wave 3 (*n* = 2992); (3) Participants who had a stroke or lacked data on stroke during the exposure period (*n* = 444 and *n* = 110, respectively); (4) Participants with extreme values of height, weight, and waist circumference (outside mean ± 3 SD) (*n* = 307); (5) Participants lacking stroke data during the outcome period (*n* = 2). Ultimately, 6179 participants were included in the final sample, among whom 489 developed incident stroke, as shown in Figure 1.

### 2.2. Exposures and Outcomes

The C-index was calculated using the formula proposed by Valdez [7,17]:

$$C\text{-}\mathrm{index}=0.109^{-1}\mathrm{WC}\left(m\right){\left[\left(\frac{\mathrm{weight}\left(\mathrm{kg}\right)}{\mathrm{height}\left(m\right)}\right)\right]}^{-\frac{1}{2}}$$

Following previously reported calculation methods for cumulative triglyceride–glucose index (TyG) [18], TyG-BMI [19], and cumulative blood pressure [20], we applied the trapezoidal integration method to calculate the cumulative C-index:

cumulative C-index = [(C-index_2011_ + C-index_2013_)/2] × (2013 − 2011) +  [(C-index_2013_ + C-index_2015_)/2] × (2015 − 2013).

The unit of the cumulative C-index was m^3/2^ kg^−1/2^ year. The cumulative C-index represents the area under the curve for the time-varying C-index within the 2011–2015 observation window; a higher value indicates a greater cumulative burden of central obesity during the observation period.

We defined new-onset stroke as the study endpoint. Based on the official CHARLS questionnaire and previous studies, we evaluated stroke status using the questionnaire items: “Have you ever received a stroke diagnosis from a physician?” and “Have you been diagnosed with stroke by a doctor since the last follow-up visit?”. Person-time was calculated from the Wave 3 (2015) baseline until the stroke event or the last follow-up, whichever came first. The timing of stroke events was determined by participants’ responses to the questionnaire items: “When was the first time you were diagnosed with or learned you had a stroke?” and “When did you have your most recent stroke?” If participants self-reported the exact time of stroke diagnosis in the questionnaire, this date was taken as the event time. If precise stroke onset data were unavailable, we assigned the event time as the midpoint between the two interview dates bracketing the stroke. For censored participants without stroke during follow-up, the date of their last completed follow-up interview was used as the censoring time [21,22].

### 2.3. Covariates

We collected demographic information, medical history, vital signs, and laboratory measurements as described in the CHARLS questionnaire [16]. (1) Demographic covariates: age, sex, residence (urban or rural), marital status (married or other), educational level (illiterate, primary school and below, junior high school, senior high school or above), and smoking and drinking status (never, current smoker/drinker, former smoker/drinker). Alcohol drinking groups were defined as follows: never drinkers reported no alcohol intake or <1 drinking occasion per month at baseline; current drinkers consumed alcohol >1 time per month and had not quit; former drinkers previously drank >1 time per month but had abstained for ≥3 months. (2) Medical history: Hypertension was defined as blood pressure (BP) ≥140/90 mmHg across all three survey measurements [23]. Diabetes mellitus was defined as glycated hemoglobin (HbA_1_c) ≥6.5% or fasting blood glucose (FBG) ≥126 mg/dL (7.0 mmol/L) [24]. Given the Chinese study population and substantial ethnic variations in lipid profiles, dyslipidemia was defined according to the 2023 Chinese Guidelines for Lipid Management: triglycerides (TG) ≥ 200 mg/dL (2.3 mmol/L), total cholesterol (TC) ≥ 240 mg/dL (6.2 mmol/L), high-density lipoprotein cholesterol (HDL-C) ≤ 40 mg/dL (1.0 mmol/L), and/or low-density lipoprotein cholesterol (LDL-C) ≥ 160 mg/dL (4.1 mmol/L) [25]. In addition, self-reported hypertension, diabetes mellitus, and dyslipidemia (including current use of relevant medications) were also considered diagnostic. Heart disease was defined as a self-reported physician diagnosis or current treatment for heart disease, including heart attacks, coronary heart disease, angina, congestive heart failure, or other heart conditions [26]. Chronic kidney disease (CKD) was defined as either a self-reported physician diagnosis of CKD (excluding malignancies) or an estimated glomerular filtration rate (eGFR) <60 mL/min/1.73 m^2^ calculated using the CKD-EPI 2021 formula [27,28]. (3) Participants’ vital signs (BP, pulse rate) and laboratory results, including hemoglobin (HGB), blood urea nitrogen (BUN), serum creatinine (Scr), uric acid (UA), FBG, C-reactive protein (CRP), TG, TC, HDL-C, LDL-C, and HbA_1_c, were collected.

### 2.4. Statistical Analysis

Table S1 summarizes missing data for the covariates in this study. To handle missing data appropriately while preserving sample information, we conducted multiple imputation. Before fitting the model, we assessed multicollinearity among variables using the generalized variance inflation factor (GVIF). For variables with multiple degrees of freedom, we examined GVIF^1/2Df^. A value ≥ 2 was considered indicative of problematic collinearity (Table S2). We then incorporated all variables listed in Table S2 into the multiple imputation model and generated 20 imputed datasets. Imputation equations were constructed using predictive mean matching (PMM) for continuous variables, logistic regression for binary variables, and multinomial logistic regression for unordered categorical variables. We evaluated model convergence using trace plots of the chained-equation imputation process. Results across multiple imputed datasets were pooled according to Rubin’s rules [29,30]. Normally distributed continuous variables were presented as mean ± standard deviation (SD), skewed continuous variables as median and interquartile range (IQR), and categorical data as counts and percentages. We used chi-square tests, ANOVA, or Wilcoxon’s rank-sum tests for group comparisons.

We categorized participants into three groups based on tertiles of the cumulative C-index: T1 (≤5.062), T2 (>5.062 to ≤5.310), and T3 (>5.310). Cox proportional hazards regression was applied to evaluate the association between cumulative C-index and incident stroke. We created four models: (1) Model 1, the unadjusted model, estimated crude hazard ratios (HRs) and corresponding 95% confidence intervals (CIs). (2) Model 2 was adjusted for age, sex, residence, educational level, marital status, drinking status, and smoking status. (3) Model 3 included all covariates from Model 2 along with hypertension, dyslipidemia, diabetes, CKD, and heart disease. (4) Model 4 further adjusted for CRP, HGB, and UA in addition to Model 3. Prior studies have indicated that metabolic conditions (hypertension, diabetes, dyslipidemia) and inflammation may mediate the association between obesity and stroke [31,32]. Including these potential mediators in the primary model would introduce over-adjustment and underestimate the total effect of the C-index on stroke. Accordingly, we specified Model 2 as the primary analytical model. Model 3 and Model 4 were constructed as supplementary analyses to evaluate the attenuation of effect estimates after further adjustment for metabolic diseases and selected laboratory biomarkers. We assessed the proportional hazards assumption using Schoenfeld residuals.

Based on three-wave C-index data from 2011 to 2015, we applied group-based trajectory modeling (GBTM) to identify longitudinal trajectories of the C-index over time. Censored normal models with 2–6 trajectories were fitted with linear, quadratic, and cubic polynomial specifications. Considering that the Bayesian information criterion (BIC) systematically favors more complex models in trajectory modelling and cannot independently determine the optimal number of trajectories, multiple diagnostic metrics were integrated for model selection (Table S3): (1) consideration of BIC and Akaike Information Criterion (AIC) fit statistics; (2) each trajectory group accounting for no less than 5% of the total sample; (3) average posterior probability (APP) >0.70 for each group; (4) overall model entropy approaching 1; and (5) the visual distinguishability and clinical interpretability of trajectory curves. Compared with the four-trajectory solution, the five-trajectory model showed lower APP values and reduced overall entropy (0.81 to 0.79). Moreover, the additional trajectory in the five-trajectory model was visually indistinguishable from adjacent trajectories and lacked meaningful clinical interpretation. Accordingly, the four-trajectory model for the C-index was selected as the optimal model. This four-trajectory model was specified with quadratic polynomials (Table S4), and the quadratic coefficients for all trajectories were statistically significant (all *p* < 0.001) [33,34]. We used Cox proportional hazards regression models to calculate the HRs and 95% CIs for stroke across different trajectory groups.

Subgroup analyses were performed to assess the consistency of associations between cumulative C-index and C-index trajectories and stroke, stratified by baseline age (<60 and ≥60 years), sex, residence, educational level, smoking history, drinking history, and medical history. We conducted several sensitivity analyses to assess the robustness of our results. First, we analyzed the complete-case sample after excluding participants with missing data. Additionally, we analyzed the association between the cumulative C-index and stroke among participants without baseline stroke risk factors (diabetes mellitus, hypertension, dyslipidemia, and smoking history). We used R software (version 4.4.1) for statistical analyses.

## 3. Results

### 3.1. Baseline Features of Participants

Among the 6179 participants, the cumulative C-index had a mean of 5.18 (SD, 0.31) and a median of 5.18 (IQR, 4.99–5.38). Table S5 shows the baseline characteristics of study participants. Compared with participants in the lower C-index tertiles, those in the higher tertiles were more likely to be female, urban residents, non-smokers and non-drinkers, and were more likely to have a higher prevalence of hypertension, diabetes, dyslipidemia and heart disease. Moreover, with SBP <140mmHg as the cutoff, the proportion of participants achieving the SBP target decreased as the cumulative C-index increased. To assess potential selection bias, we compared baseline characteristics between included and excluded participants (Table S6). The two groups differed in several characteristics, especially regarding lifestyle factors and metabolic conditions.

**Figure 2:**
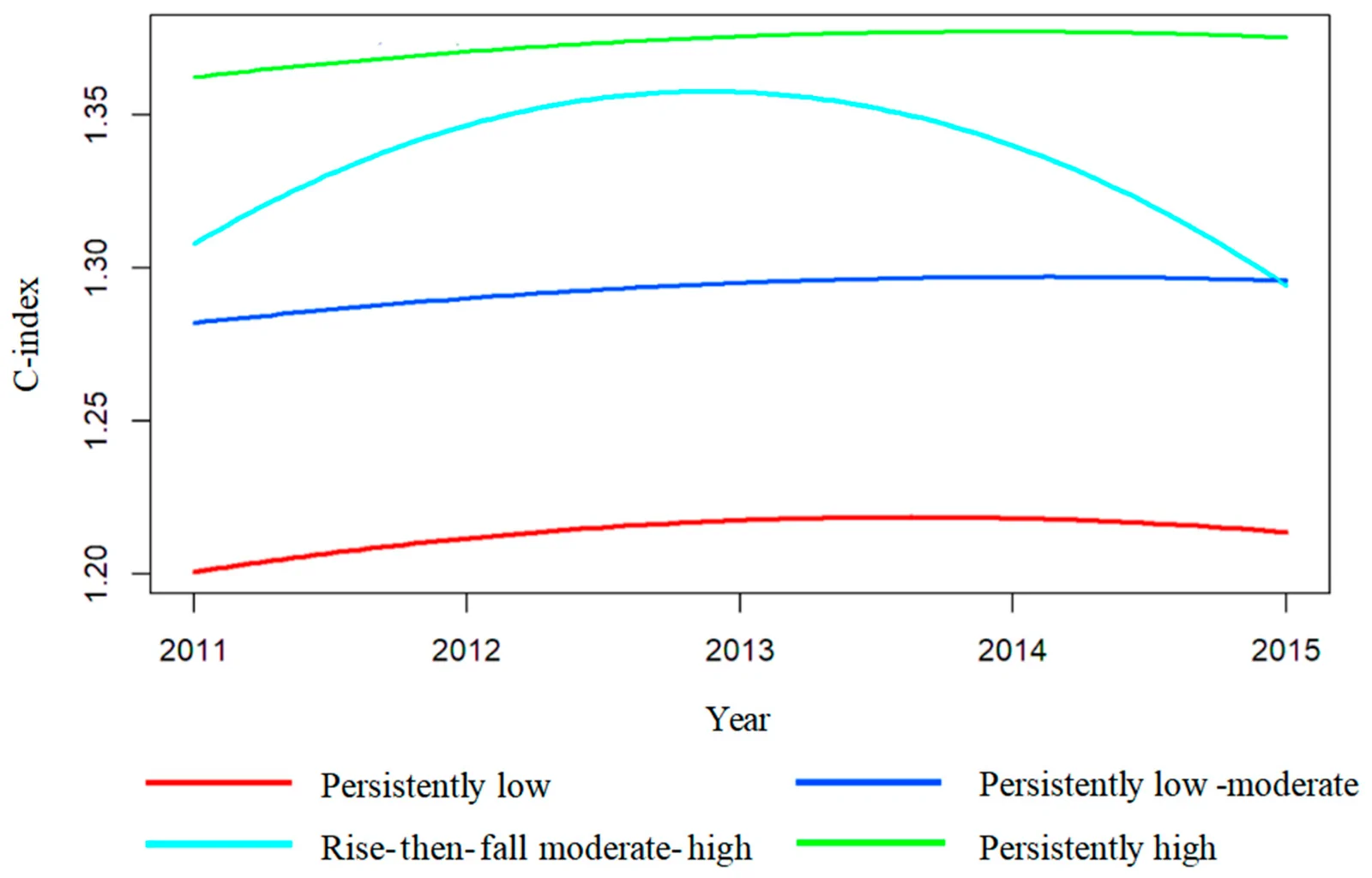
Four longitudinal trajectory patterns of the C-index.

Figure 2 illustrates four longitudinal trajectory patterns of the C-index: Class 1, “Persistently low” (*n* = 1312, 21.2%); Class 2, “Persistently low–moderate” (*n* = 2844, 46.0%); Class 3, “Rise-then-fall moderate–high” (*n* = 592, 9.6%); and Class 4, “Persistently high” (*n* = 1431, 23.2%). Baseline characteristics across the trajectory subgroups are presented in Table S7. From Class 1 to Class 4, the proportions of female participants and individuals with hypertension, diabetes, and heart disease progressively increased, as did stroke incidence. In addition, participants with a persistently high C-index had higher BP, pulse rate, and LDL-C.

### 3.2. Association of Cumulative C-Index and C-Index Trajectories with Stroke

During a median follow-up of 4.6 years, 489 (7.91%) participants developed stroke. After adjustment for demographic and lifestyle-related variables, compared with participants in the lowest tertile of the cumulative C-index (T1), those in the T2 and T3 groups had a 28% higher risk (HR = 1.28, 95% CI: 1.02–1.63, *p* = 0.040) and a 72% higher risk of stroke (HR = 1.72, 95% CI: 1.37–2.16, *p* < 0.001), respectively. After further adjustment for metabolic conditions and laboratory markers, the association between cumulative C-index and stroke remained significant, although the hazard ratios were attenuated (Table 1). This attenuation may partly reflect adjustment for mediators, as metabolism mediates part of the C-index’s effect on the risk of stroke. Schoenfeld residual tests showed no violation of the proportional hazards assumption (*p* > 0.05) (Table S8).

For different C-index trajectories, the persistently high C-index group had an 80% higher risk of stroke than the persistently low C-index group (HR = 1.80, 95% CI: 1.36–2.39, *p* < 0.001). After further adjustment for metabolic conditions and laboratory markers, a persistently high C-index remained associated with stroke (Table 2). Schoenfeld residual tests showed no violation of the proportional hazards assumption (*p* > 0.05) (Table S9).

In addition, Pearson correlation analysis showed that the Wave 1 C-index was moderately positively correlated with the cumulative C-index (r = 0.670, 95% CI: 0.657–0.684, *p* < 0.001). We further evaluated the association between the single Wave 1 C-index and stroke (Table S10). In the main model (Model 2), participants in the highest tertile of baseline C-index (T3) had a 55% higher risk of stroke compared with those in the lowest tertile (T1) (HR = 1.55, 95% CI: 1.23–1.95, *p* < 0.001). This effect size was smaller than the risk estimates for the highest tertile of the cumulative C-index and for the persistently high C-index trajectory group. Notably, this comparison only reflects differences in association strength and does not indicate superior predictive performance for the cumulative C-index. Moreover, the analytical sample in this study was restricted to participants with complete C-index measurements across all three waves (Wave 1, Wave 2, Wave 3). Individuals with only baseline Wave 1 C-index but missing Wave 2 or Wave 3 measurements were excluded during sample screening. Therefore, the findings for the baseline C-index are applicable only to the study sample, and caution is warranted when extrapolating these results.

### 3.3. Subgroup Analyses and Sensitivity Analyses

We stratified subgroup analyses by participants’ demographic information and medical history (Tables S11 and S12). No statistically significant interactions were detected for the associations of cumulative C-index and C-index trajectories with stroke across any stratifying variables (all *p*-values for interaction >0.05). Therefore, differences in statistical significance among some subgroups do not support effect modification. In addition, several subgroups had limited sample sizes, reducing statistical power. Therefore, these subgroup analyses were exploratory only and cannot be used for risk stratification.

After excluding participants with missing data, we reanalyzed the association between the cumulative C-index and stroke and eventually included 6158 participants. There were no substantial changes in the results (Table S13). For individuals free of established stroke risk factors (diabetes, hypertension, dyslipidemia, smoking history) at baseline, the cumulative C-index remained associated with the risk of stroke (Table S14). However, this screened “healthy” subgroup may differ substantially from the original study population, and the relevant results should be interpreted with caution.

## 4. Discussion

Globally, stroke remains the second leading cause of death and the third leading cause of disability. The pathogenesis of stroke is complex and is influenced by multiple risk factors, including hypertension, diabetes, smoking, air pollution, etc. Obesity is a risk factor whose contribution to stroke increased most rapidly from 1990 to 2019 [35]. It is considered an important modifiable risk factor. In recent years, researchers have paid increasing attention to body fat distribution. In particular, the C-index may be valuable for stroke risk assessment.

This study investigated a nationally representative cohort of 6179 Chinese adults. The findings revealed that participants in the T2 and T3 groups had a markedly elevated risk of stroke compared with those in the lowest tertile (T1) of the cumulative C-index. In addition, a persistently high C-index was strongly associated with an increased risk of stroke. These results are consistent with previous studies and highlight the importance of obesity and body fat distribution in stroke [15,36]. A study from the Rural China Cohort Study demonstrated the association between the C-index and the risk of ischemic stroke, showing that the C-index may predict ischemic stroke [15]. However, it relied on only a single-time-point measurement of the C-index. A single measurement cannot reflect long-term obesity-related risk. This limitation matters especially for middle-aged and older adults, in whom obesity and body fat distribution may change over time, leading to diverse health outcomes. The C-index may fluctuate considerably in some individuals, so a single measurement may fail to capture underlying risks. In contrast, cumulative metrics can better characterize long-term obesity exposure. GBTM clusters participants with similar C-index trajectories and identifies inter-individual differences in C-index change patterns [37]. Accordingly, we conducted analyses using cumulative C-index and C-index trajectories. This analytical strategy can provide a more comprehensive understanding of the obesity–stroke association and has meaningful clinical implications.

Studies have shown that obesity is associated with an elevated risk of stroke. First, visceral adipose tissue is metabolically active. It secretes multiple inflammatory mediators, vasoactive molecules, and leptin. These substances may impair vascular endothelium function and contribute to atherosclerotic plaque formation [38,39]. Second, abdominal obesity and insulin resistance constitute a vicious cycle associated with worsening lipid disorders and endothelial injury. This cycle is linked to an elevated risk of diabetes and hypertension, two independent risk factors for stroke [31,32]. Third, excess visceral fat promotes the release of free fatty acids. This may contribute to fat accumulation in the liver and pancreas, accompanied by lipid peroxidation, insulin resistance, systemic inflammation, and thrombosis [40,41]. In addition, obese individuals exhibit reduced nitric oxide bioavailability, which may impair vasodilatory capacity and endothelial anticoagulant function [42,43]. Abdominal fat distribution also serves as a useful indicator of cerebrovascular risk, potentially reflecting abnormalities in central autonomic regulation and local metabolic effects of adipose tissue [44]. Limitations of this study include: (1) Stroke and comorbidity data in this study were derived from self-reported answers in the CHARLS questionnaire. We did not have access to hospital records, ICD codes, or imaging data. Therefore, we could not objectively ascertain stroke events. Recall bias and outcome misclassification may exist. Also, we could not distinguish stroke from hemorrhagic stroke. As a result, we could not determine whether the C-index was associated with stroke across stroke subtypes. (2) We excluded participants who were lost to follow-up or lacked anthropometric data. Demographic characteristics, adiposity levels, baseline disease burden, and risk of stroke may differ in the excluded population from those in the included sample. The included participants may be healthier and more adherent to follow-up than the original cohort, indicating potential selection bias. Because many excluded participants had missing outcome data and multiple covariates required for weight estimation, we could not generate stable, reliable weights. Therefore, inverse probability weighting (IPW) was not adopted in this study. This type of selection bias cannot be eliminated, and caution should be exercised when generalizing the findings of this study. (3) Our study was based on the CHARLS cohort of middle-aged and older Chinese adults. The cutoffs for the cumulative C-index and the obesity trajectory patterns apply only to this population. The results may not directly extend to younger people or other ethnic groups. Differences in lifestyle and healthcare across regions also limit generalization to other countries. (4) We did not carry out a full head-to-head comparison of predictive performance. The cumulative C-index combines repeated measurements at several time points. It reflects long-term obesity load. This differs from a single BMI or waist circumference measurement, so a direct comparison is not appropriate. (5) We fitted the GBTM model using only three time-points (Wave 1 to Wave 3). Given the limited number of time points, the model cannot capture complex trajectory patterns well. Accordingly, the four derived trajectory classes are empirical groupings within this observation window and should not be regarded as lifelong or long-term stable obesity phenotypes. Caution is therefore warranted when interpreting trajectory-related results. (6) Some participants lacked the exact timing of stroke onset. We assigned the midpoint of the two follow-up interviews as the event time. This handling of interval-censored data may lead to bias in the effect estimates. (7) Finally, physical activity and medication use were not included as covariates. Therefore, we could not fully rule out residual confounding by these factors.

## 5. Conclusions

Overall, this study demonstrated that a high cumulative C-index and persistently elevated C-index trajectories were strongly associated with an increased risk of self-reported incident stroke among Chinese middle-aged and older adults. Future investigations should further explore the utility of the C-index in public health strategies, differentiate stroke subtypes, and facilitate effective stroke prevention.

## Figures and Tables

**Table 1:** Association between cumulative C-index and stroke.

Cumulative C-Index	Model 1		Model 2		Model 3		Model 4	
	HR (95% CI)	*p*-Value	HR (95% CI)	*p*-Value	HR (95% CI)	*p*-Value	HR (95% CI)	*p*-Value
T1	Reference		Reference		Reference		Reference	
T2	1.31 (1.03–1.66)	0.028	1.28 (1.02–1.63)	0.040	1.14 (0.90–1.45)	0.290	1.13 (0.89–1.44)	0.320
T3	1.82 (1.45–2.27)	<0.001	1.72 (1.37–2.16)	<0.001	1.35 (1.06–1.70)	0.013	1.34 (1.06–1.69)	0.014
*p* for trend		<0.001		<0.001		0.011		0.016

Note: Model 1: unadjusted; Model 2: adjusted for age, sex, marital status, residence, education level, drinking status, and smoking status; Model 3: adjusted for all covariates in Model 2 plus hypertension, diabetes, heart disease, dyslipidemia, and chronic kidney disease; Model 4: adjusted for all covariates in Model 3 plus C-reactive protein, uric acid, and hemoglobin. Abbreviations: HR, hazard ratio; CI, confidence interval.

**Table 2:** Association between C-index trajectories and stroke.

C-Index Trajectories	Model 1		Model 2		Model 3		Model 4	
	HR (95% CI)	*p*-Value	HR (95% CI)	*p*-Value	HR (95% CI)	*p*-Value	HR (95% CI)	*p*-Value
Persistently low	Reference		Reference		Reference		Reference	
Persistently low–moderate	1.30 (1.00–1.69)	0.051	1.28 (0.98–1.67)	0.069	1.10 (0.85–1.44)	0.467	1.10 (0.84–1.43)	0.493
Rise-then-fall moderate–high	1.63 (1.15–2.31)	0.007	1.54 (1.08–2.19)	0.018	1.29 (0.90–1.85)	0.158	1.29 (0.90–1.84)	0.165
Persistently high	1.92 (1.45–2.53)	<0.001	1.80 (1.36–2.39)	<0.001	1.35 (1.01–1.80)	0.043	1.34 (1.01–1.80)	0.044

Note: Model 1: unadjusted; Model 2: adjusted for age, sex, marital status, residence, education level, drinking status, and smoking status; Model 3: adjusted for all covariates in Model 2 plus hypertension, diabetes, heart disease, dyslipidemia, and chronic kidney disease; Model 4: adjusted for all covariates in Model 3 plus C-reactive protein, uric acid, and hemoglobin. Abbreviations: HR, hazard ratio; CI, confidence interval.

## Data Availability

The dataset supporting the conclusions of this article is available in the China Health and Retirement Longitudinal Study (CHARLS) repository (https://charls.pku.edu.cn, accessed on 15 October 2025).

## Supplementary Materials

The following supporting information can be downloaded at: https://www.mdpi.com/article/10.3390/healthcare14213741/s1. Table S1: Distribution of variables with missing data; Table S2: Collinearity diagnostics for covariates; Table S3: Results of Group-Based Trajectory Modeling (GBTM) clustering; Table S4: The final four-group trajectory model of C-index; Table S5: Basic characteristics of the participants—according to cumulative C-index tertiles; Table S6: Baseline characteristics of included and excluded participants; Table S7: Basic Characteristics of the participants—according to C-index trajectories; Table S8: Proportional hazards test for cumulative C-index and stroke; Table S9: Proportional hazards test for C-index trajectories and stroke; Table S10: Association between C-index measured at Wave 1 and stroke; Table S11: Subgroup analysis of the association between cumulative C-index and stroke; Table S12: Subgroup analysis of the association between C-index trajectories and stroke; Table S13: Association between cumulative C-index and stroke after excluding individuals with missing values; Table S14: Association between cumulative C-index and stroke after excluding individuals with definite stroke risk factors at baseline.

## Abbreviations

The following abbreviations are used in this manuscript:

C-index	Conicity index
BMI	Body mass index
BRI	Body roundness index
ABSI	A body shape index
RFM	Relative fat mass
WHtR	Waist-to-height ratio
WC	Waist circumference
CHARLS	China Health and Retirement Longitudinal Study
TyG	Triglyceride–glucose index
HbA_1_c	Glycated hemoglobin
FBG	Fasting blood glucose
TG	Triglycerides
TC	Total cholesterol
HDL-C	High-density lipoprotein cholesterol
LDL-C	Low-density lipoprotein cholesterol
CKD-EPI	Chronic kidney disease epidemiology collaboration
eGFR	Estimated glomerular filtration rate
BP	Blood pressure
HGB	Hemoglobin
BUN	Blood urea nitrogen
Scr	Serum creatinine
UA	Uric acid
CRP	C-reactive protein
GVIF	Generalized variance inflation factor
PMM	Predictive mean matching
SD	Standard deviation
IQR	Interquartile range
HR	Hazard ratio
CI	Confidence interval
GBTM	Group-based trajectory modeling
BIC	Bayesian information criterion
AIC	Akaike information criterion
